# Atracurium Besylate and other neuromuscular blocking agents promote astroglial differentiation and deplete glioblastoma stem cells

**DOI:** 10.18632/oncotarget.6314

**Published:** 2015-11-13

**Authors:** Raffaella Spina, Dillon M. Voss, Laura Asnaghi, Andrew Sloan, Eli E. Bar

**Affiliations:** ^1^ Department of Neurological Surgery, Case Western Reserve University School of Medicine and Case Comprehensive Cancer Center, Cleveland, OH, USA; ^2^ Department of Pathology, Johns Hopkins University, School of Medicine, Baltimore, MD, USA; ^3^ Department of Neurological Surgery, University Hospital-Case Medical Center, Case Comprehensive Cancer Center, and Case Western Reserve University, Cleveland, OH, USA

**Keywords:** Atracurium Besylate, stem cells, glioma, astrocytic differentiation, neurotransmitter signaling

## Abstract

Glioblastoma multiforme (GBM) are the most common primary malignant brain tumor in adults, with a median survival of about one year. This poor prognosis is attributed primarily to therapeutic resistance and tumor recurrence after surgical removal, with the root cause suggested to be found in glioblastoma stem cells (GSCs). Using glial fibrillary acidic protein (GFAP) as a reporter of astrocytic differentiation, we isolated multiple clones from three independent GSC lines which express GFAP in a remarkably stable fashion. We next show that elevated expression of GFAP is associated with reduced clonogenicity *in vitro* and tumorigenicity *in vivo*. Utilizing this *in vitro* cell-based differentiation reporter system we screened chemical libraries and identified the non-depolarizing neuromuscular blocker (NNMB), Atracurium Besylate, as a small molecule which effectively induces astroglial but not neuronal differentiation of GSCs. Functionally, Atracurium Besylate treatment significantly inhibited the clonogenic capacity of several independent patient-derived GSC neurosphere lines, a phenomenon which was largely irreversible. A second NNMB, Vecuronium, also induced GSC astrocytic differentiation while Dimethylphenylpiperazinium (DMPP), a nicotinic acetylcholine receptor (nAChR) agonist, significantly blocked Atracurium Besylate pro-differentiation activity. To investigate the clinical importance of nAChRs in gliomas, we examined clinical outcomes and found that glioma patients with tumors overexpressing CHRNA1 or CHRNA9 (encoding for the AChR-α1 or AChR-α9) exhibit significant shorter overall survival. Finally, we found that *ex-vivo* pre-treatment of GSCs, expressing CHRNA1 and CHRNA9, with Atracurium Besylate significantly increased the survival of mice xenotransplanted with these cells, therefore suggesting that tumor initiating subpopulations have been reduced.

## INTRODUCTION

Glioblastoma multiforme (GBM) is the most common and aggressive primary malignancy of the central nervous system in adults [1]. There is still no curative treatment for this disease and most patients succumb to disease around 14 months post diagnosis [2, 3]. One potential reason for this dismal prognosis may be the fact that GBM are extremely heterogeneous in regards to their cellular, genetic, epigenetic, and molecular make-up [4]. Therefore, more effective therapies are urgently needed, including treatments which can target the subpopulation of cancer stem cells (CSCs) thought to be resistant to current standard of care therapy [5–7].

First identified in acute myeloid leukemia [8], cancer stem cells (CSCs), also referred to as tumor-initiating/propagating cells [9], have been isolated from a variety of solid tumors using a wide array of markers [10–14]. CSCs are therefore defined by their stem cell characteristics and tumor initiation and propagation ability [13, 15].

Accumulating data suggests that neural network activity controls proliferation and/or differentiation of normal neural stem cells (NSCs) in the adult brain. To this end, choline acetyltransferase (ChAT) [16], gamma-aminobutyric acid (GABA), and nitric oxide (NO)-releasing neurons [17] in the SVZ, as well as serotonergic neurons [18] in the raphe nucleus have all been demonstrated to promote NSC proliferation in the SVZ via direct synaptic contact with NSCs and/or ependymal cells. For decades it was generally believed that nicotinic AChRs (nAChRs) only exist in the nervous system (neuronal nAChRs) and at neuro-muscular junctions (muscle mAChRs). However, more recent work clearly showed that nAChRs and their physiological agonist, acetylcholine, are universally expressed in mammalian cells, including cancer cells [19]. They act as central regulators of a complex network of stimulatory and inhibitory neurotransmitters that govern the synthesis and release of growth, angiogenic and neurogenic factors in cancer cells and their microenvironment, and in distant organs. In addition, nAChRs stimulate intracellular signaling pathways in a cell-type-specific manner.

In this study we took a slightly different approach to studying GSCs. We focused on their differentiated progenies (e.g., cells expressing astroglial and neuronal markers). Following isolation of multiple subclones from three independent patient-derived neurosphere models we found that GFAP expression, as defined by flow cytometric analysis, was remarkably stable over multiple passages *in vitro*, suggesting that tumor heterogeneity is maintained by subpopulations defined by GFAP expression. In addition, we found that subclones expressing low levels of GFAP (GL = GFAP Low) were more clonogenic *in vitro* and tumorigenic *in vivo* in comparison with their cellular counterparts which express high levels of GFAP (GH = GFAP High). Finally, we report on the identification of Atracurium Besylate, a non-depolarizing neuromuscular blocker, as a drug capable of inducing astroglial differentiation of GSC. We show that Atracurium Besylate-induced astrocytic differentiation is associated with reduced clonogenicity *in vitro* and reduced capacity to initiate intracranial xenografts *in vivo.* Mechanistically, Atracurium Besylate appears to promote GSC astrocytic differentiation through AChRs as Vecuronium, another neuromuscular blocker, has similar pro-differentiation effects. Taken together, our results suggest that pharmacological manipulation of AChR, and potentially their downstream signaling pathways, may promote the conversion of aggressive GSC into less aggressive, more differentiated counterparts.

## RESULTS

### Glial fibrillary acidic protein (GFAP) marks an astroglial-like subpopulation of GBM cells with reduced clonogenic and tumorigenic capacities *in vitro* and *in vivo*, respectively

We, as well as others, have previously reported that GFAP expression is increased following treatments which directly target GSCs [20–22]. In these studies we showed that inhibition of Hedgehog and or Notch signaling also resulted in reduced clonogenic capacity *in vitro* and xenograft engraftment and progression *in vivo*. However, direct evaluation of human GBM cultures originated from individual cells which express endogenously high or low levels of GFAP have never been reported. Furthermore, it is not clear if these subsets differ in their capacity to initiate or maintain tumors. Based on our previous work, we hypothesized that more differentiated cells, expressing high levels of GFAP, would be less clonogenic *in vitro* and may give rise to less aggressive tumors or even fail to form tumor xenografts *in vivo* as compared to less differentiated cells which express low levels of GFAP.

**Figure 1:**
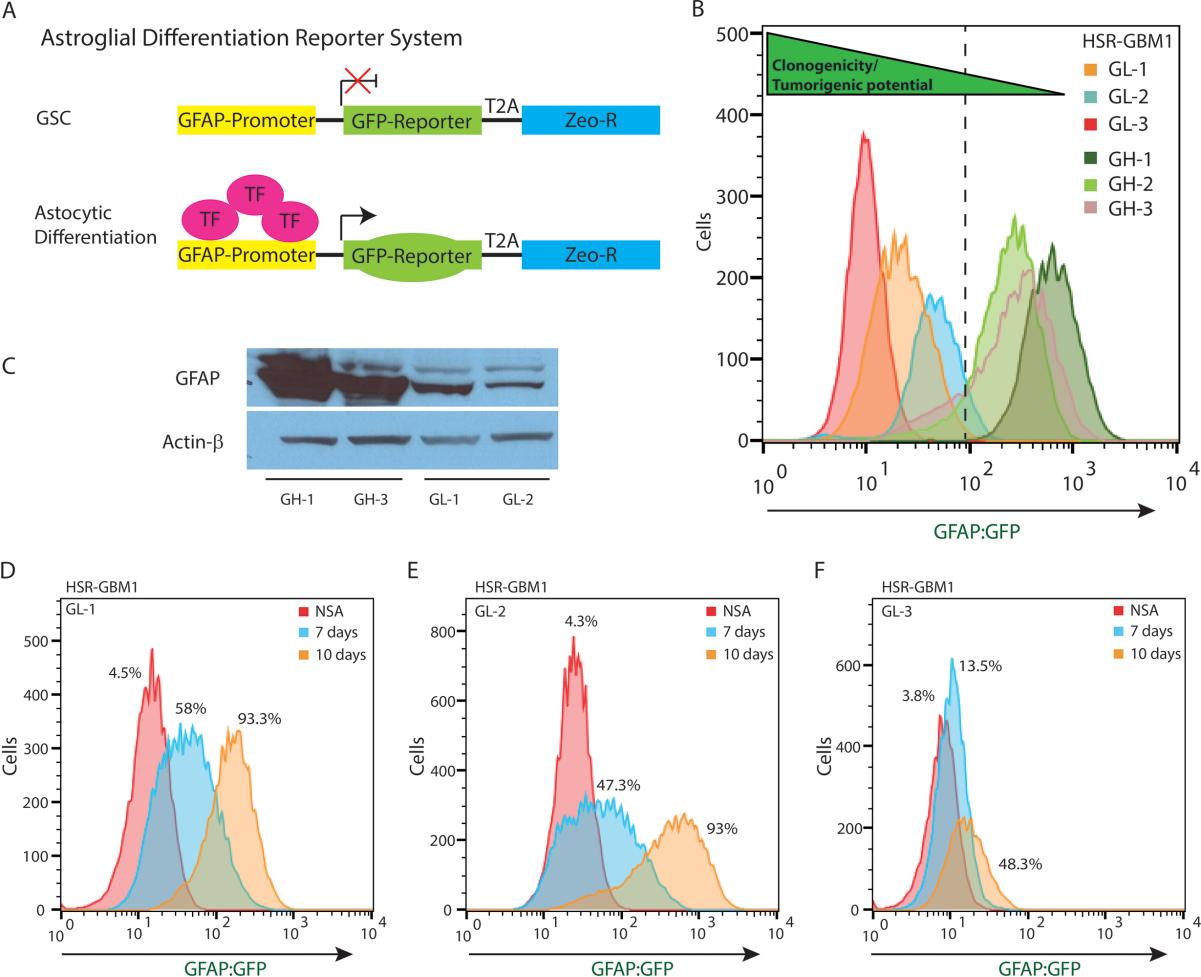
pGreenZeo GFAP:GFP Reports Astroglial Differentiation in GSC (**A**) Schematic diagram of pGreenZeo GFAP:GFP reporter construct. GSC, which are found in an undifferentiated state, do not have the appropriate combination of transcription factors necessary to bind to the glial fibrillary acidic protein (GFAP) promoter and therefore will not express GFP (top panel). In contrast, upon differentiation into astrocytes, transcription factors that bind to the GFAP promoter, will be expressed resulting in transcription of GFP and the cells will fluoresce green (bottom panel). (GSC - Glioma stem cell, T2A-protein linker, Zeo-R - Zeocin resistance gene, TF - transcription factor). (**B**) HSR-GBM1 was transduced with GFAP:GFP reporter lentivirus and multiple subclones were selected based on GFP expression in the neurosphere initiating-cell and confirmed by flow cytometry. These clones were named either GL (GFAP Low) or GH (GFAP High). (**C**) Western blot analysis confirming HSR-GBM1 GH subclones express high levels of GFAP protein as compared with their GL counterparts. (**D**–**F**) Upon removal of growth factors and addition of fetal calf serum to the growth medium, HSR-GBM1 subclones GL-1, GL-2 and GL-3 differentiate as indicated by a significant and time-dependent increase in the percentage of cells expressing the GFAP:GFP reporter. (NSA – neural stem cell medium).

To monitor GSC astroglial differentiation, we transduced HSR-GBM1, HSR040622, and HSR040821 neurospheres with pGreenZeo lenti-reporters encoding for a green-fluorescent protein (GFP) fused in-frame with a Zeocin resistance cassette driven by the human GFAP promoter-element (Figure 1A–SBI, Mountain View, CA). We next isolated individual clones followed by flow cytometric determination of the percentage of cells expressing GFP. Neurosphere clones, derived from single cells, containing ≤ 5% GFP-positive cells are referred to as GL (GFAP Low) while clones composed of ≥ 75% GFP-positive cells are referred to as GH (GFAP High) and are considered to be more differentiated as compared to the GL subclones (Figure 1B). Western blot analysis confirmed that GFP percentage, determined by flow cytometry, corresponded with endogenous levels of GFAP protein, with *GH* subclones exhibiting higher levels of GFAP protein compared to their *GL* counterparts (Figure 1C). To further validate our astroglial differentiation system, we next induced astroglial differentiation using an established differentiation protocol [23] and measured the magnitude of GFAP:GFP reporter induction. We found that parental (not shown) as well as all three *GL* subclones showed significant induction of the reporter with *GL-1* (Figure 1D) GFAP:GFP percentage increasing from 4.5% when cells are cultured under standard neural stem-cell growth conditions to 58% and 93.3% seven and ten days post induction of differentiation, respectively. Similar increases were found in *GL-2* (Figure 1E) (4.3% to 47.3% and 93%) and *GL-3* (Figure 1F) (3.8% to 13.5% and 48.3%). We have previously shown that expression of the Notch pathway direct targets, Hes1 and Hes5, are significantly elevated in GSCs [22, 24]. We therefore determined Notch targets expression in HSR-GBM1 GL and GH subclones and normalized expression levels to those found in HSR-GBM1 GH-1. We found that all three HSR-GBM1 GL lines had significantly elevated levels of Hes1 mRNA (Supplementary Figure S1A). Hes5 levels were elevated in HSR-GBM1 GL-2 and HSR-GBM1 GL-3 subclones but not in HSR-GBM1 GL-1 or any of the three HSR-GBM1-GH subclones (Supplementary Figure S1B). Finally, expression of the third Notch target, Hey2, was significantly elevated in the HSR-GBM1 GL-1 and GL-2 subclones (Supplementary Figure S1C). Taken together this set of experiments suggests that the Notch pathway is active in cells expressing low levels of GFAP and confirm that Notch is downregulated upon astroglial differentiation of GSCs.

**Figure 2:**
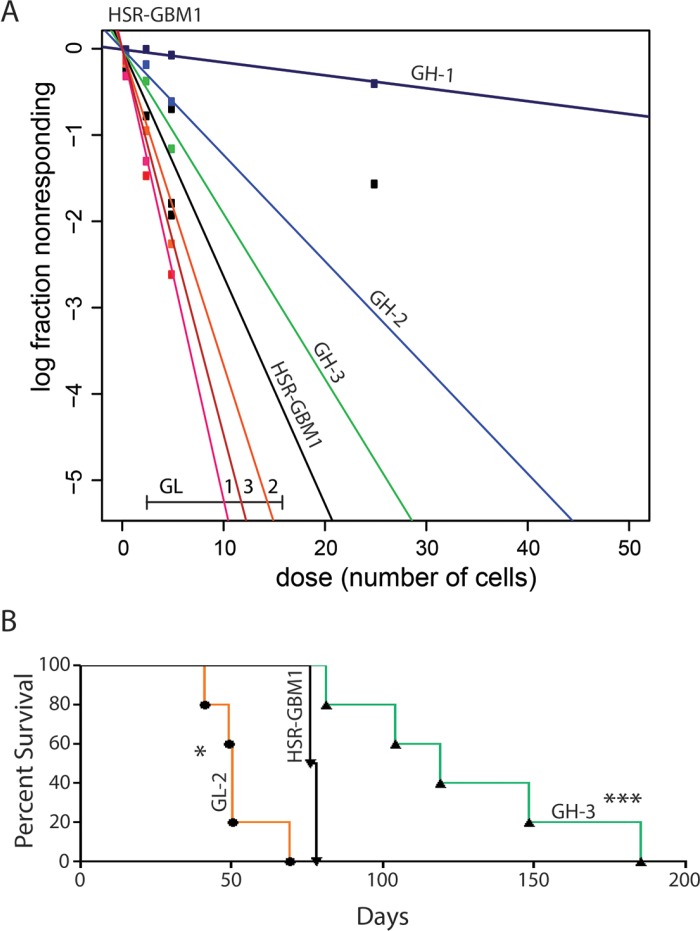
Functional characterization of HSR-GBM1 GFAP:GFP subclones (**A**) HSR-GBM1 GL subclones are more clonogenic *in vitro* as indicated by increased GSC frequencies which are measured by an Extreme Limiting Dilution Analysis (ELDA). (**B**) In addition to being more clonogenic *in vitro*, HSR-GBM1 GL subclones are also more tumorigenic *in vivo* as indicated by reduced survival of mice implanted with HSR-GBM1 GL-2 as compared with the parental HSR-GBM1 line or with HSR-GBM1 GH-3. (*p < 0.05, ***p < 0.0001, Log-rank (Mantel-Cox Test), respectively); HSR-GBM1 *n* = 2, HSR-GBM1 GH and GL subclones, *n* = 5.

To test our hypothesis that *GL* subclones are more clonogenic than their *GH* counterparts we employed an *in vitro* clonogenic assay termed extreme limiting dilution assay or ELDA [25]. This assay is based on the notion that GSCs are functionally defined by their ability to form neurospheres from a single cell *in vitro* [26]. We therefore plated *GH* and *GL* subclones at increasing cell densities and evaluated neurosphere formation nine days later. We found that stem cell frequencies were 1/133.4 for *GH-1* (*p* = 5.5 × 10^−107^), 1/16.2 for *GH-2* (*p* = 9.4 × 10^−12^), 1/10.5 for *GH-3* (*p* = 0.003), all which were significantly less clonogenic than parental HSR-GBM1 cells (averaged stem cell frequency 1/7.6) and the *GL* subclones with 1/3.8 for *GL-1* (*p* = 1.7 × 10^−11^), 1/5.5 for *GL-2* (*p* = 0.0015) and 1/4.5 for *GL-3* (*p* = 2.7 × 10^−7^) (Figure 2A). Importantly, we found that the original phenotypes (e.g., percentage of GFAP:GFP and *in vitro* clonogenic capacity) of individual subclones were maintained over many passages *in vitro* (data shown in Figure 2A represents the average of three independent assays performed over 12 passages spanning 8 months). These results suggest that the *GL* subclones were originated by clonogenic GSCs while the *GH* subclones were derived from less clonogenic and potentially differentiated cells defined by their expression of the astroglial differentiation marker GFAP.

Finally, as the gold standard for defining cancer stem cells remains tumor propagation [27], we next implanted 1 × 10^5^ cells orthotopically using CD133-negative *GL-1*, *GL-2*, and the GH subclones *GH-1*, and *GH-3*. Parental HSR-GBM1 cells were also implanted and served as a control. Consistent with our hypothesis, we found that median survival for animals implanted with *GL-2* cells was significantly shorter (50 days, log rank test *p* < 0.05, Figure 2B, orange) as compared to the parental line HSR-GBM1 (77 days). In addition, we calculated median survival for animals implanted with CD133-negative *GL-1* (107 days log rank test *p* < 0.01, not shown), *GH-1* (94 days log rank test *p* < 0.01, not shown), and *GH-3* (over 119 days, log rank test *p* < 0.0001) (Figure 2B, green). Taken together, these data strongly support our hypothesis that GFAP-negative cells originally isolated from HSR-GBM1 neurospheres are true GSC or precursor cells while clones derived from GFAP-positive cells are more differentiated and significantly less tumorigenic.

### Screening for inducers of astroglial differentiation

**Figure 3:**
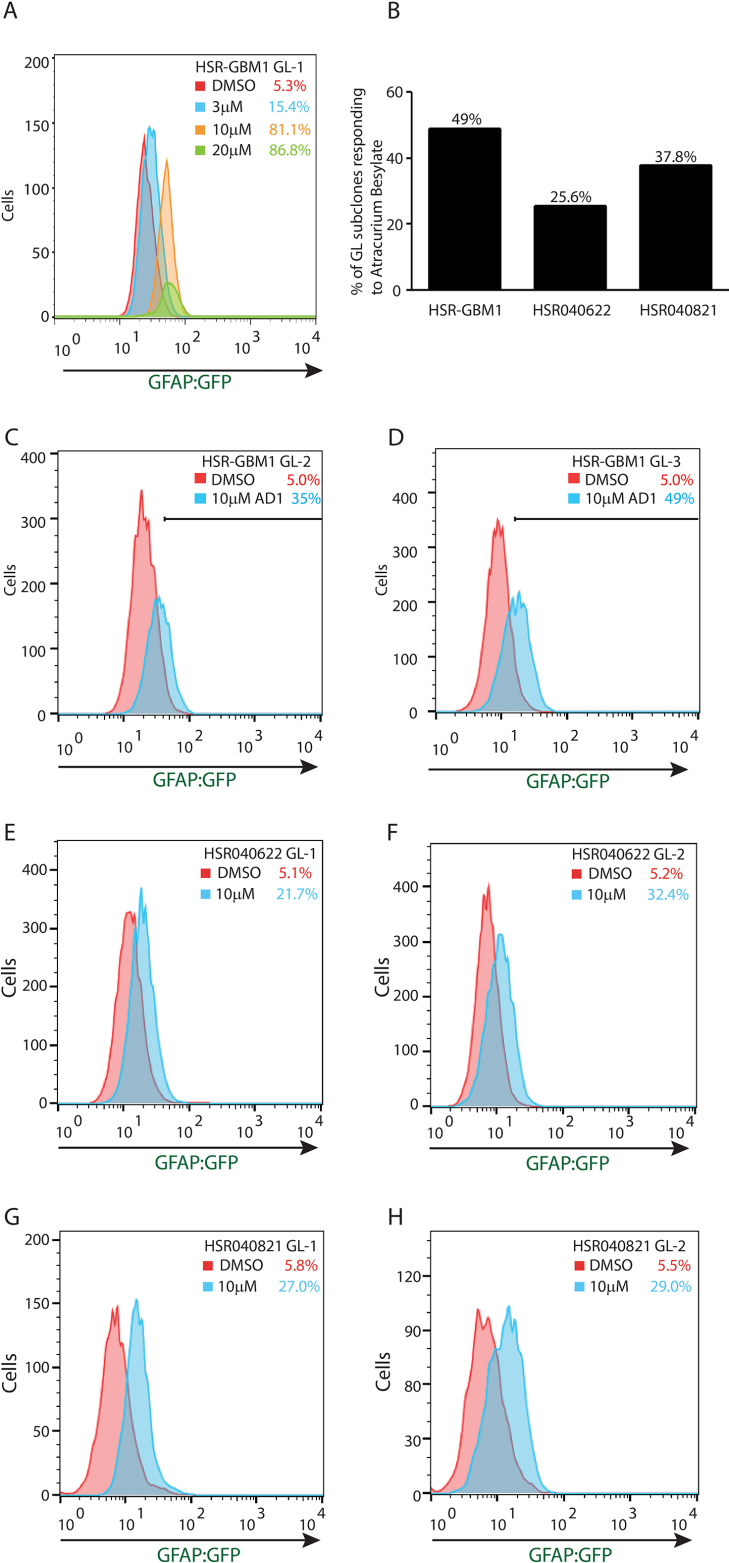
Atracurium Besylate promotes astroglial differentiation of GSCs (**A**) Flow cytometric analysis of HSR-GBM1 GL-1 treated for 72 hours with increasing concentrations of Atracurium Besylate. Treatment increased the percentage of GFP-positive cells in a dose-dependent manner from 5.3% in DMSO (red) to 15.4%, 81.1%, and 86.8% in 3 μM (blue), 10 μM (orange), and 20 μM (green) Atracurium Besylate, respectively. (**B**) Differential response to Atracurium Besylate by individual GL subclones of HSR-GBM1 (25/51 subclones (49%)), HSR040622 (11/43 subclones (25.6%)), and HSR040821 (14/37 subclones (37.8%)). (**C**–**H**) Treatment with Atracurium Besylate increases the percentage of cells expressing the GFAP-GFP reporter in multiple subclones of three independent GSC neurosphere lines tested.

In an effort to identify agents and pathways which may control astroglial differentiation in GSCs, we performed a small-molecule drug screen using two NIH Clinical Collection libraries. For this screen, we utilized *GL-1* neurospheres as they demonstrated the largest magnitude of change in our *in vitro* forced differentiation assay (Figure 1D). Cells were treated for 72 hours with library agents set at a concentration of 2 μM or equal volume of DMSO as control. Following incubation, we determined the percentage of cells expressing the GFAP-GFP reporter by flow cytometry. Baselines for viability and percentage of GFP positive cells were determined in at least three wells for each library plate and a positive hit was determined as an increase in the percentage of GFP-positive cells of three standard deviations over baseline (DMSO) and a minimum threshold of 25% GFP positive cells. We identified 12 drugs that induced sufficient increase in the GFP-positive population as set by our criteria (Supporting information (SI); Table S1). In this report we chose to focus on the non-depolarizing neuromuscular blocker, Atracurium Besylate due to its significant effects on GSC astroglial differentiation, inducing GFAP:GFP reporter from 5% to 40.8%. As shown in Figure 3A, we detected dose dependency with 3, 10, and 20 μM Atracurium Besylate increasing the percentage of GFAP:GFP expressing cells from 5.3%, in DMSO, to 15.4%, 81.1%, and 86.8%, respectively. In addition to the above mentioned properties, Atracurium Besylate, an AChR receptor antagonist, represents an attractive lead compound modulating neurotransmitter signaling, which has just been very recently linked to clonal chemo-response in GBM [28].

### Atracurium Besylate promotes astroglial but not neuronal differentiation

We next examined the effects of Atracurium Besylate on several additional GL subclones of HSR-GBM1 and of two additional GSC lines, HSR040622 and HSR040821, integrating the GFAP-GFP reporter cassette. Cells were treated for 72 hours with Atracurium Besylate or vehicle (DMSO), as control, and the percentage of GFP-positive cells was determined by flow cytometry. We found that treatment resulted in significant increases in astroglial differentiation in all GSC lines we tested. In regards to individual subclones, we found that 49% (25 of 51 subclones) of HSR-GBM1, 25.6% (11 of 43 subclones) of HSR040622, and 37.8% (14 of 37 subclones) of HSRGBM040821 responded significantly to Atracurium Besylate (Figure 3B). Selected individual subclones are shown in Figure 3C–3H. HSR-GBM1 GL-2 increasing from 5.0% to 35.0% (Figure 3C), HSR-GBM1 GL-3 increasing from 5.0% to 49.0% (Figure 3D), HSR040622 GL-1 increasing from 5.1% to 21.7% (Figure 3E), HSR040622 GL-2 increasing from 5.2% to 32.4% (Figure 3F), HSR040821 GL-1 increasing from 5.8% to 27.0% (Figure 3G), and HSR040821 GL-2 increasing from 5.5% to 29.0% (Figure 3H). These data suggest that Atracurium Besylate promotes astroglial differentiation, albeit with different magnitude, in multiple independent GBMs.

To determine if Atracurium Besylate specifically promotes astroglial differentiation we next examined its effects on neuronal differentiation. To this end, we transduced HSR-GBM1 GSC line with a neuronal differentiation reporter system (System Biosciences) where GFP expression is driven by the promoter of late neuronal differentiation marker, microtubule associated protein-2 (MAP-2). Several HSR-GBM1 MAP2-Low (ML) subclones were established and treated with a high dose of Atracurium Besylate for 72 hours. We could not detect significant changes in MAP2-GFP percentage following Atracurium Besylate treatment (individual analysis of HSR-GBM1 ML-1 is shown in Supplementary Figure S2A and the averaged results of three independent subclones: ML-1, ML-2, and ML-3 is shown in Supplementary Figure S2B). These data suggest that Atracurium Besylate promotes GSC differentiation towards an astroglial but not neuronal cell fate.

### Atracurium Besylate induced astroglial differentiation is associated with reduced GSC frequency

**Figure 4:**
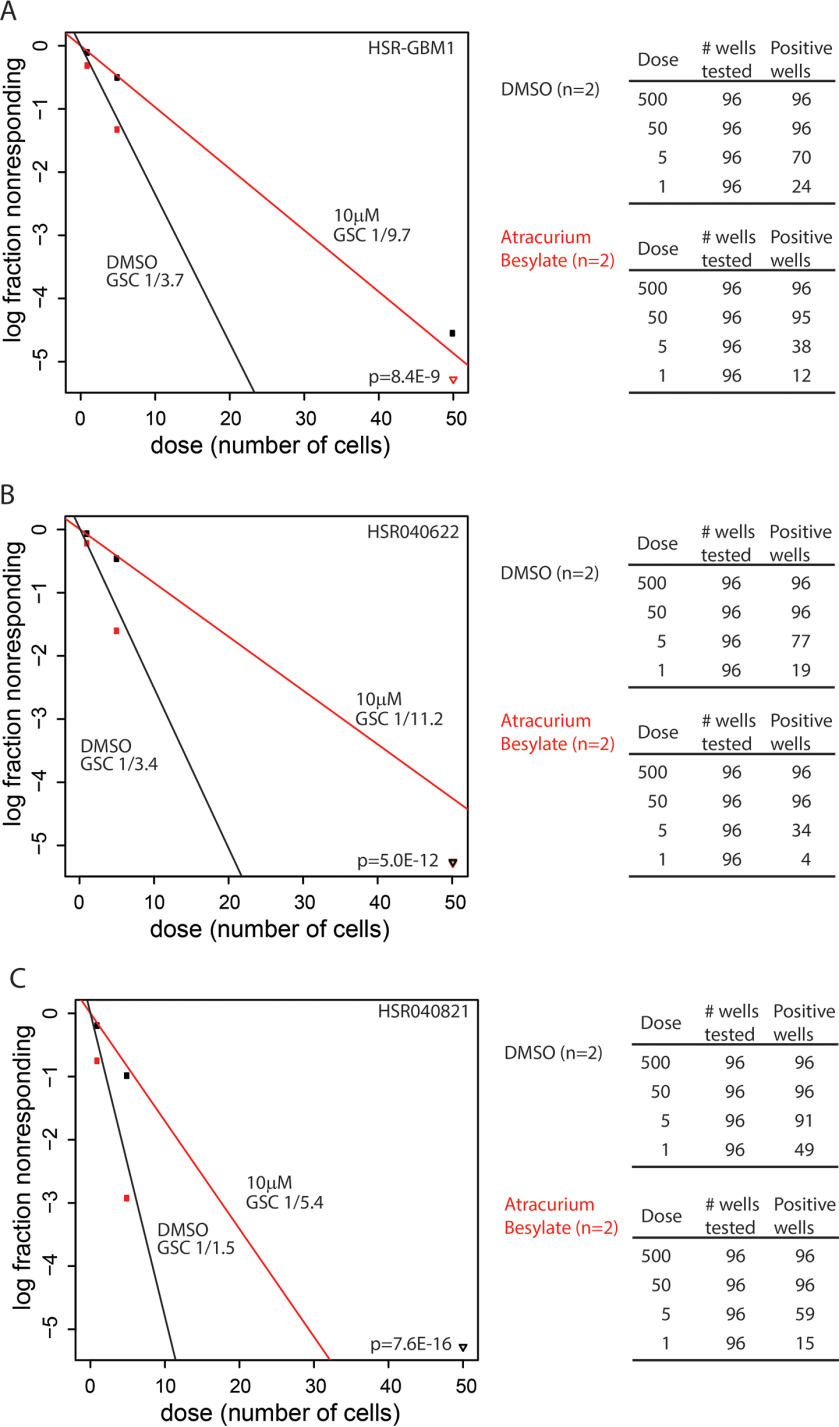
Atracurium Besylate reduces clonogenic frequencies *in vitro* HSRGBM-1 (**A**), HSR040622 (**B**), and HSR040821 (**C**) GSC lines were treated with 10 μM Atracurium Besylate (red) or DMSO (black) as control. Treatment lasted 48 hours and was followed by a drug wash-off and recovery for an additional 24 hours. Clonogenic frequencies were determined by an ELDA (see materials and methods). Stem cell frequencies are indicated below each label. Limiting dilution analyses were performed in triplicate and *p*-values indicated on each graph. Detailed data for wells tested are shown on the right of each plot.

We have previously reported that treatments which target GSCs result in increased GFAP expression and reduced clonogenic and tumorigenic capacities of GSCs [21, 22]. To directly measure GSC frequencies, we utilized the extreme limiting dilution assay method developed by Hu et al. [25]. We found that 48 hours treatment with 10 μM Atracurium Besylate, followed by a drug wash-off and recovery period for 24 hours, significantly inhibited GSC frequencies in the three lines we tested. In HSR-GBM1 Atracurium Besylate reduced GSC frequency from 1 in 3.7 to 1 in 9.7 (*n* = 2; Figure 4A, *p* = 8.4 × 10^−9^; detailed data shown to the right); In HSR040622 from 1 in 3.4 to 1 in 11.2 (*n* = 2; Figure 4B, *p* = 5.0 × 10^−12^; detailed data shown to the right), and in HSR040821 from 1 in 1.5 to 1 in 5.40 (*n* = 2; Figure 4C, *p* = 7.6 × 10^−16^; detailed data shown to the right). Collectively, these data suggest that reduced clonogenic frequency, mediated by Atracurium Besylate, may be a result of induced astroglial differentiation of GSC and therefore might prevent tumor engraftment.

### Atracurium Besylate reduces tumor engraftment and increases survival of mice xenotransplanted with *ex-vivo* treated GSCs

**Figure 5:**
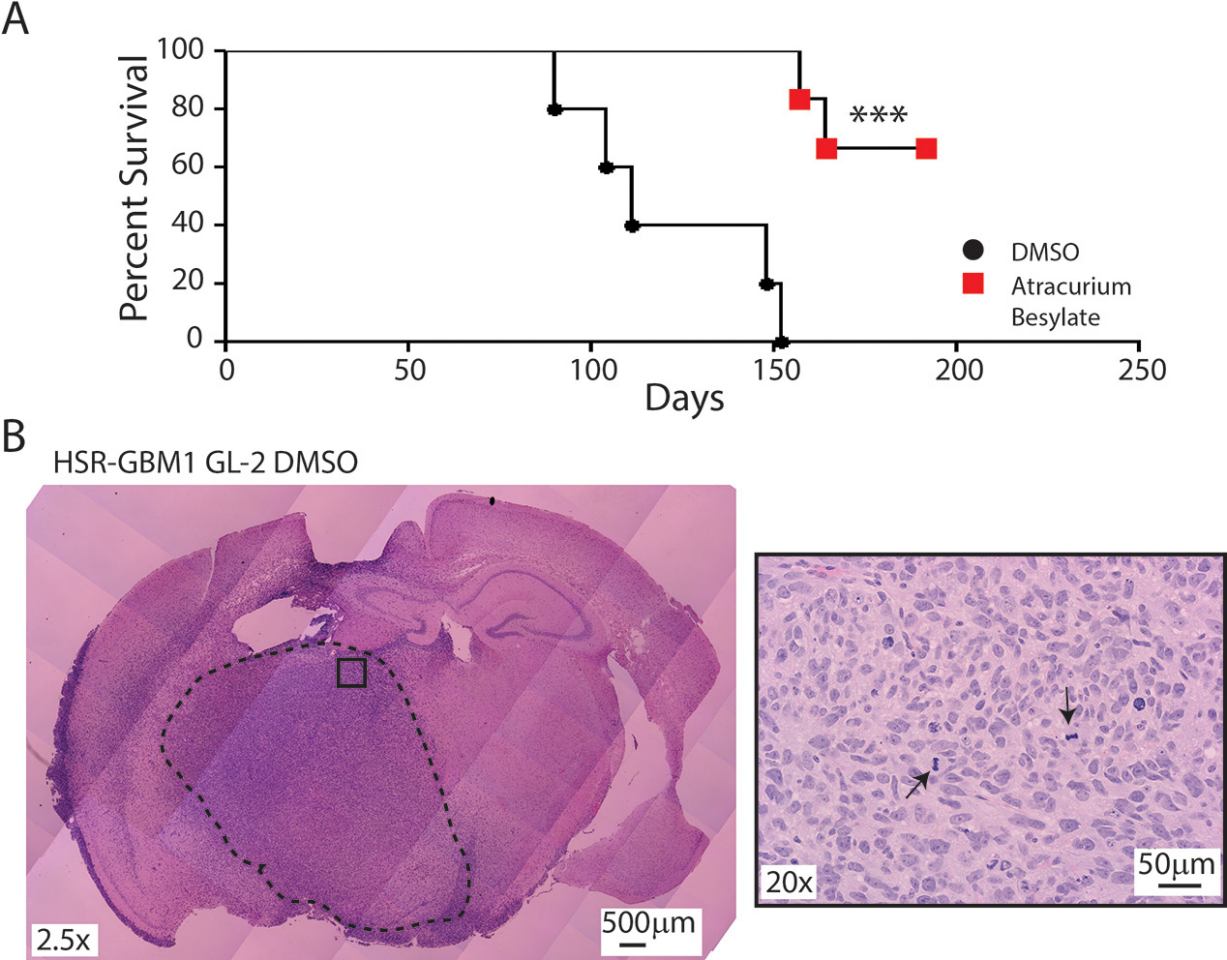
Atracurium Besylate inhibits tumor engraftment *in vivo* (**A**) 5 of 5 HSR-GBM1 GL-2 cultures pretreated with DMSO engrafted when 10,000 viable cells were injected into the brains of NOG mice. In contrast, when the same number of viable cells was injected after treatment with 10 μM Atracurium Besylate, only 2 of 6 animals engrafted. Log-rank analysis of Kaplan-Meier survival curves indicates that the prolongation of survival associated with Atracurium Besylate pretreatment is significant (***p = 0.0007). (**B**) H&E staining of brains injected with DMSO-treated HSR-GBM1 GL-2 cells showing highly proliferative and invasive tumors. Right panel shows a higher magnification (20x) of the tumor “core” with arrowheads pointing at mitotic figures.

To test this hypothesis, HSR-GBM1 GL-2 cells were treated with vehicle or 10 μM Atracurium Besylate for 48 hours in culture followed by drug wash-off and recovery for additional 24 hours before intracerebral implantation of 1 × 10^4^ viable cells (*n* = 6 animals per group). We chose to utilize HSR-GBM1 GL-2 as it represents the most aggressive subclone *in vivo*; in addition, in an attempt to increase the confidence that changes in survival are due to reduction in tumor initiating subpopulations, we reduced the number of cells inoculated in this set of experiments to one tenth of the number of cells inoculated in the clonal comparison experiments shown in Figure 2B. As shown in Figure 5A, pretreatment with Atracurium Besylate significantly inhibited tumor engraftment and growth, as 4 out of 6 animals, implanted with Atracurium Besylate treated-cells, survived for the duration of the experiment. In contrast, massive infiltrative gliomas were present in mice implanted with vehicle-treated cells, resulting in death as early as 3 months following implantation (Figure 5B).

### Atracurium Besylate induce astroglial differentiation through AChR

The nicotinic acetylcholine receptors (nAChRs) are members of a superfamily of ligand-gated ion channels that mediate fast signal transmission at synapses. However, emerging research shows that nAChRs and their physiological agonist acetylcholine are universally expressed in mammalian cells, including cancer cells [19]. Atracurium Besylate antagonizes the neurotransmitter action of acetylcholine by binding competitively to one or two alpha subunits on the post synaptic nAchRs found on the motor end-plate [29].

**Figure 6:**
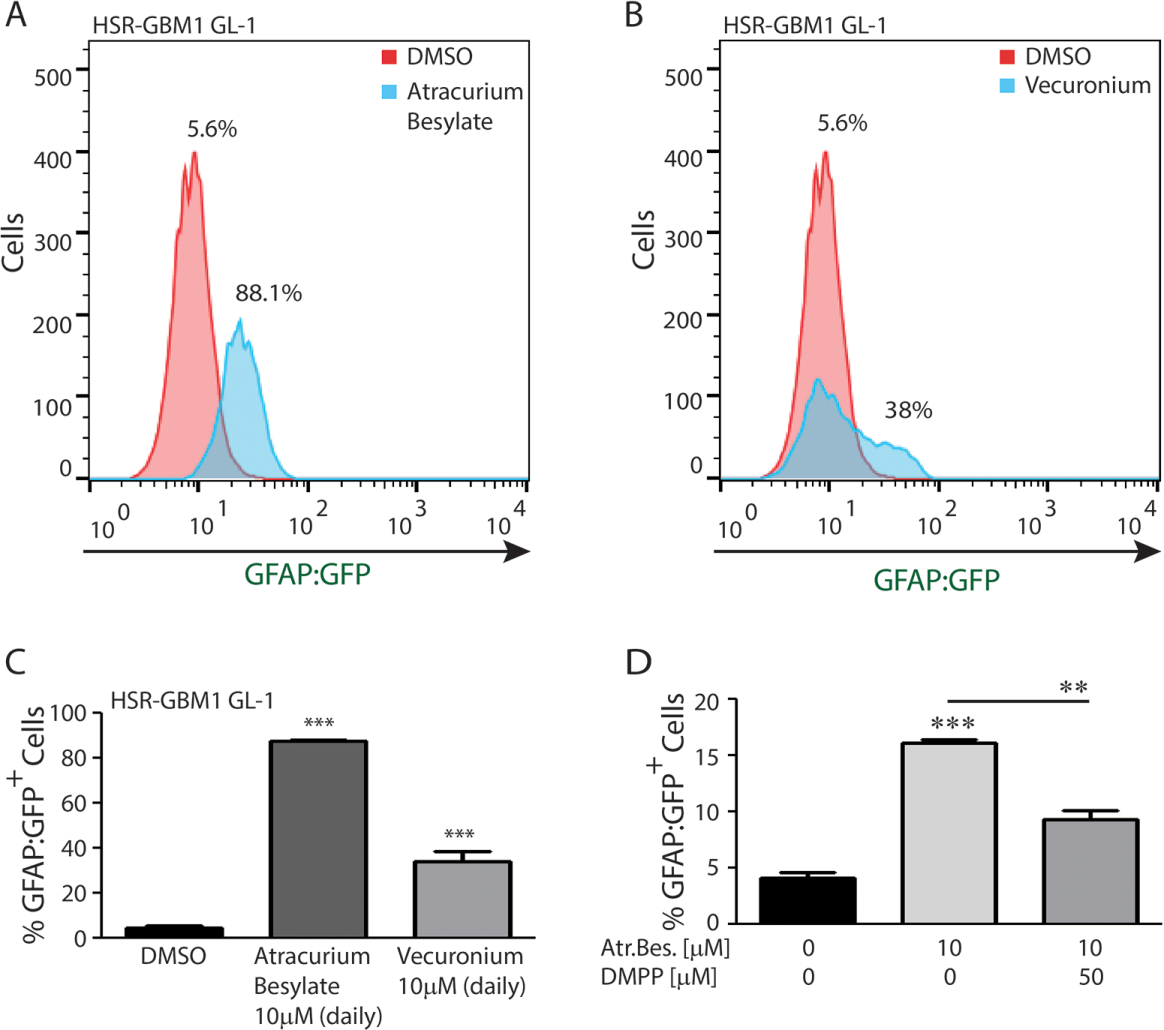
nAChR perturbations promote GSC astroglial differentiation (**A**) A representative histogram showing that daily treatment with Atracurium Besylate increases the percentage of HSR-GBM1 GL-1 cells expressing the astroglial differentiation reporter GFAP:GFP from 5.6% to 88.1%. (**B**) A representative histogram showing that treatment with Vecuronium, a second non-depolarizing agent increases the percentage GFAP:GFP positive cells from 5.6% to 38%. (**C**) Averaged results of three replicates: Percentage of GFAP:GFP positive cells increased from 4.7% in DMSO to 87.3% (***p < 0.0001, student *t*-test) and 34% (***p = 0.0005, student *t*-test) for Atracurium Besylate and Vecuronium, respectively. (**D**) Averaged results of three replicates: In HSR-GBM1 GL2 cells, Atracurium Besylate treatment significantly increased the percentage of GFAP:GFP positive cells from 4.7% in DMSO to 16.15% (***p < 0.0001, student *t*-test). This effect was significantly reduced by DMPP as only 9.27% (**p < 0.01, student *t*-test) of the cells were positive in the double-treated cultures.

To test if Atracurium Besylate may promote GSC astroglial differentiation through AChR, we treated HSR-GBM1 GL-1 with Vecuronium which is another non-depolarizing neuromuscular blocker. Like Atracurium Besylate, Vecuronium also binds to nAChRs on the motor endplate and blocks access to the receptors. Since Vecuronium is highly labile in aqueous solutions, we used a daily treatment schedule in this set of experiments. HSR-GBM1 GL-1 cells treated with DMSO contained 5.6% GFP-positive cells (Figure 6A, red histogram). When cells were treated daily for 72 hours with 10 μM Atracurium Besylate, the percentage of GFP-positive cells increased up to 88.1% (Figure 6A, blue histogram). Daily treatment with 10 μM Vecuronium increased the percentage of GFP-positive cells from 5.6% in DMSO (Figure 6B, red histogram) up to 38% (Figure 6B, blue histogram). The average of triplicates is shown in Figure 6C. To further substantiate the proposed mechanism of Atracurium Besylate action we next attempted to block its pro-differentiation activity using the nAChR agonist Dimethylphenylpiperazinium (DMPP). We found that a single dose of Atracurium Besylate more than tripled the percentage of cells expressing the GFAP:GFP reporter (from 4.13% in cultures treated with vehicle to 16.15% in cultures treated with 10 μM Atracurium Besylate). Treatment with DMPP partially but significantly blocked Atracurium Besilate activity as only 9.27% of cells were expressing the reporter in cultures co-treated with 10 μM Atracurium Besylate and 50 μM DMPP (Figure 6D). Taken together, these sets of data suggest that the AChR may be intimately involved in controlling GSC cell fate and that perturbation of AChR, using Atracurium Besylate or other neuromuscular blockers, preferentially promote astroglial differentiation.

### AChRa1 and AChRα9 are overexpressed in some gliomas and their expression level is inversely correlated with patient survival

Since Atracurium Besylate is known to inhibit AChRs by competitive binding to their alpha subunits, we next investigated the potential clinical relevance of AChRα subunit expression levels in gliomas. To this end, we utilized the Rembrandt portal (https://caintegrator.nci.nih.gov/rembrandt/) to correlate glioma patient survival with CHRNA expression (encoding for one of nine known AChR-α subunits). This analysis revealed that survival of patients with tumors overexpressing CHRNA1 or CHRNA9, which are both expressed in all our GSC lines (Supplementary Figure S2), is significantly shorter as compared to all gliomas (Supplementary Figure S3 A,B; *n* = 343). In addition, using The Cancer Genome Atlas (TCGA) data set on Oncomine (https://www.oncomine.org), we found that CHRNA1 and CHRNA9 are both significantly overexpressed in GBM with 1.65 and 2.45 fold increase over normal, respectively (Supplementary Figure S3C, S3D). Collectively, these data suggest that some AChR alpha subunits, which may be targeted by Atracurium Besylate, are clinically relevant as they are overexpressed in GBM and their expression level is inversely correlated with patient survival.

## DISCUSSION

The challenges inherent in developing more effective treatments for malignant gliomas include their relentless invasiveness, resistance to standard treatments, genetic complexity and molecular adaptability, and a subpopulation of GBM cells with phenotypic similarities to normal stem cells (GSC). New therapies will therefore be required to achieve a better outcome in patients affected by these aggressive tumors. Unfortunately, although a transient response to standard therapy is seen in the majority of patients, essentially all malignant gliomas, particularly GBM, rapidly recur. It is thought that the recurrence of GBM following current therapies is due to the persistent presence of GSCs within tumors that are relatively resistant to cell death (reviewed in [30]). Further support to this notion is provided by the elegant work of Meyer and colleagues who demonstrated that individual clones within a single tumor show unique molecular signatures, proliferation and differentiation capacities, and drug responsiveness [28].

One approach our group, as well as others, has been studying in the past several years is aimed at promoting astroglial differentiation or death of GSCs. For example, Piccirillo and coworkers elegantly showed that BMP4 protein promote astroglial differentiation of GSCs *in vitro* and *in vivo* [20]. We have recently shown that GFAP expression is increased following Hedgehog [21] or Notch [22] blockade in GSCs *in vitro* and *in vivo*. While most previous studies have focused on GSC and the markers which define them, in this study we decided to take the reverse approach. We focus primarily on the differentiated progenies of GSCs (e.g., cells expressing astroglial and neuronal markers). In addition, given the enormous cellular, expression, genetic, and epigenetic heterogeneities found in GBM, we believe an important component of our studies is the examination of a large number of subclones (up to 96 in some cases) of several independent patient-derived neurosphere lines. These allowed us, for the first time, to realize that GFAP expression, as defined by flow cytometric analysis, was remarkably stable over multiple passages *in vitro*. These observations suggest that tumor heterogeneity may be maintained by relatively stable subpopulations defined by GFAP expression. We suggest that epigenetic and / or genetic differences between individual subclones dictate the relative distribution of GFAP high/low subpopulations, potentially through regulation of symmetric versus asymmetric cell divisions, and that these differences have significant bearing on the pathobiology of individual clones. Further studies will be required to fully understand the mechanisms controlling these processes.

Using a cell-based screen, utilizing our astroglial differentiation reporter system and the NIH Clinical Collection libraries, we have identified the non-depolarizing neuromuscular blocking agent, Atracurium Besylate, which antagonizes the action of the neurotransmitter acetylcholine by competitively binding to one of the two alpha subunits on the post synaptic nAChR. The present study is the first to report that non-depolarizing neuromuscular blocking agents such as Atracurium Besylate and Vecuronium induce astroglial differentiation of GSCs and that the mechanism by which these agents work is likely to be inhibition of AChRs. Atracurium Besylate treatment dramatically reduces clonogenicity of GSCs *in vitro* and significantly increases the survival of animals implanted with *ex-vivo* treated GSCs. We believe the pro differentiation effects of Atracurium Besylate are likely to be irreversible as xenograft-derived neurospheres maintained reporter expression even after prolonged passaging *in vitro* (not shown).

The clinical relevance of AChRs in glioma is further supported by the survival and expression analyses performed using the NCI-Rembrandt and the Cancer Genomic Atlas (TCGA) data sets on Oncomine, respectively. We identified CHRNA1 and CHRNA9, two alpha subunits of the AChR complex, to be upregulated in glioma with strong association between expression and patient survival.

Our cell-based compound screen allowed us to identify different molecular mechanisms and new potential candidates for GBM treatment that will be further investigated. Interestingly, Digoxin, one of the compounds identified in the screen for its pro-differentiation effects, has been previously shown by our group and by others [31–33] to impair *in vitro* GBM invasion under hypoxia and to reduce GBM xenograft engraftment and growth of hypoxic GBM cells.

In addition to Atracurium Besylate, we identified two FDA approved calcium channel blockers: Nisoldipine and Lomerizine as potent inducers of astroglial differentiation in GSCs (Supplementary Table S1). These and similar other calcium channel blockers have been previously shown by different groups to exert an antiproliferative effect on glioma cell growth [34], enhance the chemotherapeutic effect of vincristine in an intracranially transplanted rat glioma model [35], and to inhibit DNA repair [36].

Our study uncovers a novel link between AChR and the regulation of astroglial differentiation in GBM. It also strengthen the therapeutic value of a drug-induced cancer stem cell differentiation and suggest that selective nicotinic-Acetyl Choline Receptor (nAChR) targeting as a potential novel therapeutic strategy for these universally lethal neoplasms.

## MATERIALS AND METHODS

### Ethics statement

Discomfort was minimized by the use of anesthesia during potentially painful procedures (intracranial injections). The anesthetic used was Ketamine-Xylazine in sterile saline injected intraperitoneally in accordance with the Case Western Reserve University Institutional Animal Care and Use Committee (IACUC) [Protocol number 2012–0132].

### Cell culture

The HSR-GBM1, HSR040622, and HSR040821 neurospheres were isolated by Galli and colleagues from primary glioblastoma tumors. The lines were maintained in culture as originally described [13]. Cells were counted using Guava easyCyte^™^ 5HT flow cytometer with the GUAVA Viacount reagent according to the manufacturer's instructions (Millipore Corp. Billerica, MA). All three neurosphere lines are IDH^WT^. MGMT Methylation is present in HSR-GBM1 and HSR040821. The status of MGMT methylation in HSR040622 is unknown. HSR-GBM1 is P53^WT^ while HSR040821 is P53^S278P^ [22].

### Chemicals

The NIH clinical collection libraries were purchased from Evotec (Small Molecule Repository (MLSMR) operated by Evotec (US) Inc., South San Francisco, CA). Atracurium Besylate (Catalog #SRP07340a) was purchased from Sequoia Research Products Ltd. (Pangbourne, United Kingdom) and suspended in DMSO. Vecuronium bromide (Catalog #76904) and Dimethylphenylpiperazinium (DMPP, Catalog #D9542) were purchased from Sigma and suspended in H_2_O immediately before use.

### RNA extraction and quantitative polymerase chain reaction

RNA was extracted and purified using RNeasy kit (Qiagen, Valencia, CA). Reverse transcription and quantitative PCR were performed using MultiScribe^®^ Reverse Transcriptase from ABI Systems (Applied Biosystems, Foster City, CA) and SYBR Green PCR Master Mix (Bio-Rad, Hercules, CA) on an I-Cycler IQ5 Real-Time detection system (Bio-Rad) according to the manufacturer's instructions. Expression levels were determined using the standard curve method with all expression levels being normalized to HPRT; all measurements were performed in triplicates. The following primers were used: human Hes1: forward 5′-AGTGAAGCACCTCCGGAAC-3′, reverse 5′-TCACCTCGTTCATGCACTC-3′; Hes5: forward 5′-CCGGTGGTGGAGAAGATC-3′, reverse 5′-TAGTCCTGGTGCAGGCTCTT-3′; human Hey2: forward 5′-AGATGCTTCAGGCAACAGGG-3′, reverse 5′-CAAGAGCGTGTGCGTCAAAG-3′; and human HPRT: forward 5′-CTTTGCTGACCTGCTGGATT-3′, reverse 5′-GTTGAGAGATCATCTCCACC-3′.

### Lentiviral transduction

pGreenZeo GFAP:GFP and pGreenZeo MAP2:GFP lentivirus reporter systems were purchased from SBI (System Biosciences) as a pre-packaged lentiviral preparation. GBM neurosphere cultures were plated and transduced with viruses at multiplicity of infection equal to 5 and according to manufacturer instructions. GFAP:GFP and MAP2:GFP reporter expression was verified using a Guava easyCyte 5 HT Flow Cytometer (Millipore) with at least 5,000 viable cells used for each acquisition.

### Subclone selection, expansion, and validation

Following pGreenZeo GFAP:GFP or pGreenZeo MAP2:GFP lentivirus transduction, cells were plated at a density of one cell per well in at least four 96-multiwell plates. Wells containing a single neurosphere were further examined using a Olympus IX81 fluorescent microscope for the presence of green fluorescence in at least a subset of the cells comprising the neurosphere. Following clone expansion, flow cytometry was performed to determine the exact percentage of GFP expressing cells. Clones which contained ≤ 5% GFAP:GFP positive cells were designated as GFAP Low or GL. Clones which contained ≥ 75% GFAP:GFP positive cells were designated as GFAP High or GH. Similarly, clones which contained ≤ 5% MAP2:GFP positive cells were designated as MAP2 Low or ML. Clones which contained ≥ 75% MAP2:GFP positive cells were designated as MAP2 High or MH. Parental (non-transduced) GSC lines were used to set baseline fluorescence in all experiments. Because all our clones were derived from single cells and GFP fluorescence was present in at least a subset of cells this procedure ensured that all clones had the reporter cassette integrated in all the cells and that expression of GFP therefore faithfully report GFAP (or MAP2) reporter activity.

### Western blotting

Cells were lysed in TNE (50 mM Tris-Cl (pH 7.4), 150 mM NaCl, 5 mM EDTA + Detergents = 0.5% NP40, 0.5% Deoxycholate, 1% SDS) buffer including protease inhibitor cocktail diluted 1:100 (Roche). 20 μg protein was loaded for each sample. Nitrocellulose membranes (Invitrogen) were incubated overnight at 4°C with Anti-GFAP, clone GA5 (Millipore, MAB360) at 1:1000 and β-actin, clone AC-74 (Sigma, St. Louis, MO, A5316) at 1:10,000.

### Flow cytometry

In experiments utilizing flow cytometry, GSCs were dissociated by incubation with equal volume of Accutase (Sigma) and short (usually less than 30 minutes) incubation at 37°C. Next, cultures were triturated gently to ensure single cell suspensions are achieved. Flow cytometric analysis was performed using a Guava easyCyte™ 5HT flow cytometer (Millipore). Parental (untransduced) GSC lines were used to set baseline fluorescence in all experiments and at least 5000 viable cells were acquired from each sample.

### ELDA self-renewal assays

To assess self-renewal capacity, cells were plated in 96 well plates in complete growth medium at clonal density (ranged between 5 and 500 cells per well). For drug treatment studies, cells were treated with compound or vehicle (DMSO). Neurospheres were dissociated into single-cell suspensions and plated at clonal density. Nine days after plating, plates were scored for formation of secondary neurospheres by direct visualization under a light microscope. Statistical analyses were conducted as previously described [25] using an online interface available at http://bioinf.wehi.edu.au/software/elda/index.html.

### Small molecules drug library screen

The drug library screen was done in a 96-well plate format using the NIH Clinical Collection I and II libraries (arrays of 446 and 281 small molecules, respectively) at concentration of 2 μM. Total viable cell number was determined using Guava easyCyte^™^ 5HT flow cytometer with Viacount reagent. Specific protocol details and hit candidate selection criteria are described in Supplementary Data.

### Xenograft studies

To assess *in vivo* tumorigenic capacity, a dissociated cell suspension of 1 × 10^5^ of HSR-GBM1 GL or GH subclones was stereotactically injected into the right striatum of immunodeficient mice as previously described [21]. Mice were monitored daily for neurologic changes and tumor growth, and were sacrificed when symptomatic.

For drug studies, HSR-GBM1 GL-2 cells were treated in 10 ml of media, with drug or vehicle for 48 hours. The drug was then washed off and the cells were allowed to recover for 24 hours. For xenograft studies, an aliquot collected from each flask following treatment was scored for viability by Guava easyCyte^™^ 5HT flow cytometer and Guava ViaCount Reagent (Millipore). Next, 1 × 10^4^ viable cells were injected into the right striatum of six animals per group of immunodeficient mice (NOD.Cg-Prkdcscid Il2rgtm1Wjl/SzJ (NOG)). Mice were monitored daily and sacrificed at the first indication of tumor development (ataxia, seizure, lethargy, or cachexia). One animal in the control group died two days post-surgery and thus was removed from analysis. Brains were surgically removed and fixed immediately in formalin before submission for histological analysis, as previously described [37]. All animal experimentation was done with institutional approval following NIH guidelines.

### Statistical analyses

Data are expressed as means ± SD for a minimum of three replicates. The experiments were repeated at least twice. Comparisons of mean values between groups were performed using Student's *t*-test or two way ANOVA. Significance was accepted at a value of *p* < 0.05. Comparisons of survival curves were made using the log-rank test (Kaplan-Meier). All tests were performed by using GraphPad Prism 5.0 (GraphPad Software, La Jolla, CA).

## SUPPLEMENTARY MATERIALS FIGURES AND TABLES


