# Supplementary material for: Atracurium Besylate and other neuromuscular blocking agents promote astroglial differentiation and deplete glioblastoma stem cells
Source: Oncotarget. 2015 Nov 13;7(1):459–72. doi: 10.18632/oncotarget.6314 (PMC4808011; doi:10.18632/oncotarget.6314)
Supplement: Supplementary file 1 [file oncotarget-07-0459-s001.pdf]

Atracurium Besylate and other neuromuscular blocking agents promote astroglial differentiation and deplete glioblastoma stem cells

Supplementary Materials

Supplementary Table S1: Small molecules inducing GFAP-GFP reporter expression in HSR-GBM1 GL-1

Name	Fold increase in GFP + Cells	Description	Predicted to cross Blood Brain Barrier	Drug Bank Probability
Vinorelbine	8.97	Anti-mitotic chemotherapy	−	0.88
Diphenoxylate	13.89	Antidiarrheal	+	0.96
Lomerizine	10.89	Calcium channel blocker / Cerebral vasodilator	NA	NA
Phenprobamate	10.73	Anxiolytic / Muscle relaxant, centrally acting	NA	NA
6-Azauridine	15.07	Antimetabolite / Antiviral	NA	NA
Irinotecan	14.14	Topisomerase I inhibitor	+	0.63
Atracurium Besylate	8.16	Nondepolarizing skeletal muscle relaxant	+	0.93
Glimepiride	8.75	Antidiabetic	+	0.73
Hexachlorophene	9.83	Antiseptic	+	0.92
Digoxin	8.23	Cardiotonic glycoside	−	0.72
Flecainide	10	Anti-arrhythmia agent	+	0.86
Nisoldipine	5.05	Calcium channel blocker	−	0.95

Twelve compounds were found to increase the percentage of GFAP:GFP expressing cells (fold increase is shown) and selected based on two criteria:1) Induction over 3 standard deviations over the baseline (DMSO), 2) Over 25% GFAP:GFP positive cells present at the 72 hours time point. Atracurium Besylate was selected for subsequent analyses. The ability and probability of a given agent to cross the blood brain barrier (Drug Bank (http://www.drugbank.ca/) are indicated (+/−). Abbreviation: NA (information not available).

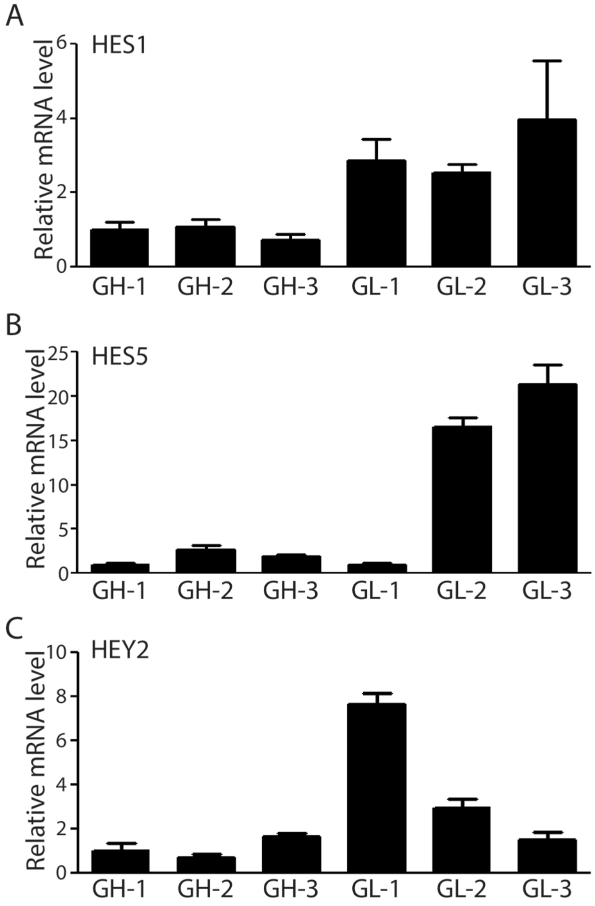

Supplementary Figure S1: Clonogenic GSC subclones overexpress Notch. mRNA levels of at least two of the three established Notch transcriptional targets, Hes1 (**A**), Hes5 (**B**), or Hey2 (**C**) are significantly elevated in undifferentiated HSR-GBM1 GL-1, GL-2, and GL-3 subclones as compared to the more differentiated subclones HSR-GBM1 GH-1, GH-2, and GH-3. mRNA levels were normalized in-sample to endogenous Hypoxanthine-guanine phosphoribosyltransferase (HPRT) and then across samples to HSR-GBM1 GH-1.

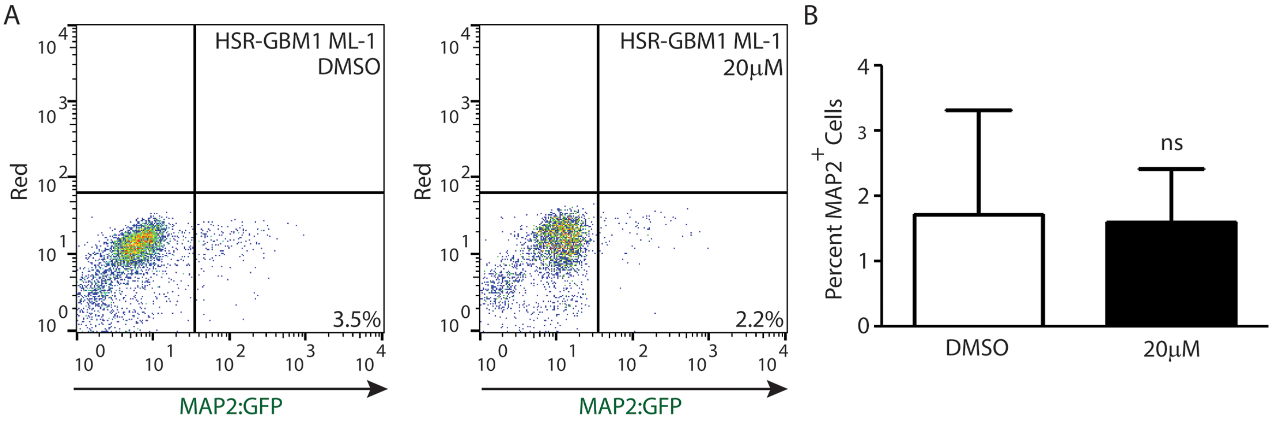

Supplementary Figure S2: Atracurium Besylate does not induce GSC neuronal differentiation. (**A**) Representative flow cytometric analysis of HSR-GBM1 ML-1 cells showing lack of induction of the MAP2:GFP reporter by Atracurium Besylate even at a high dose of 20 μM. (**B**) Combined flow cytometric analyses of three independent HSR-GBM1 ML subclones: ML-1, ML-2, and ML-3 all treated with 20 μM Atracurium Besylate for 72 hours. (ns = not significant)

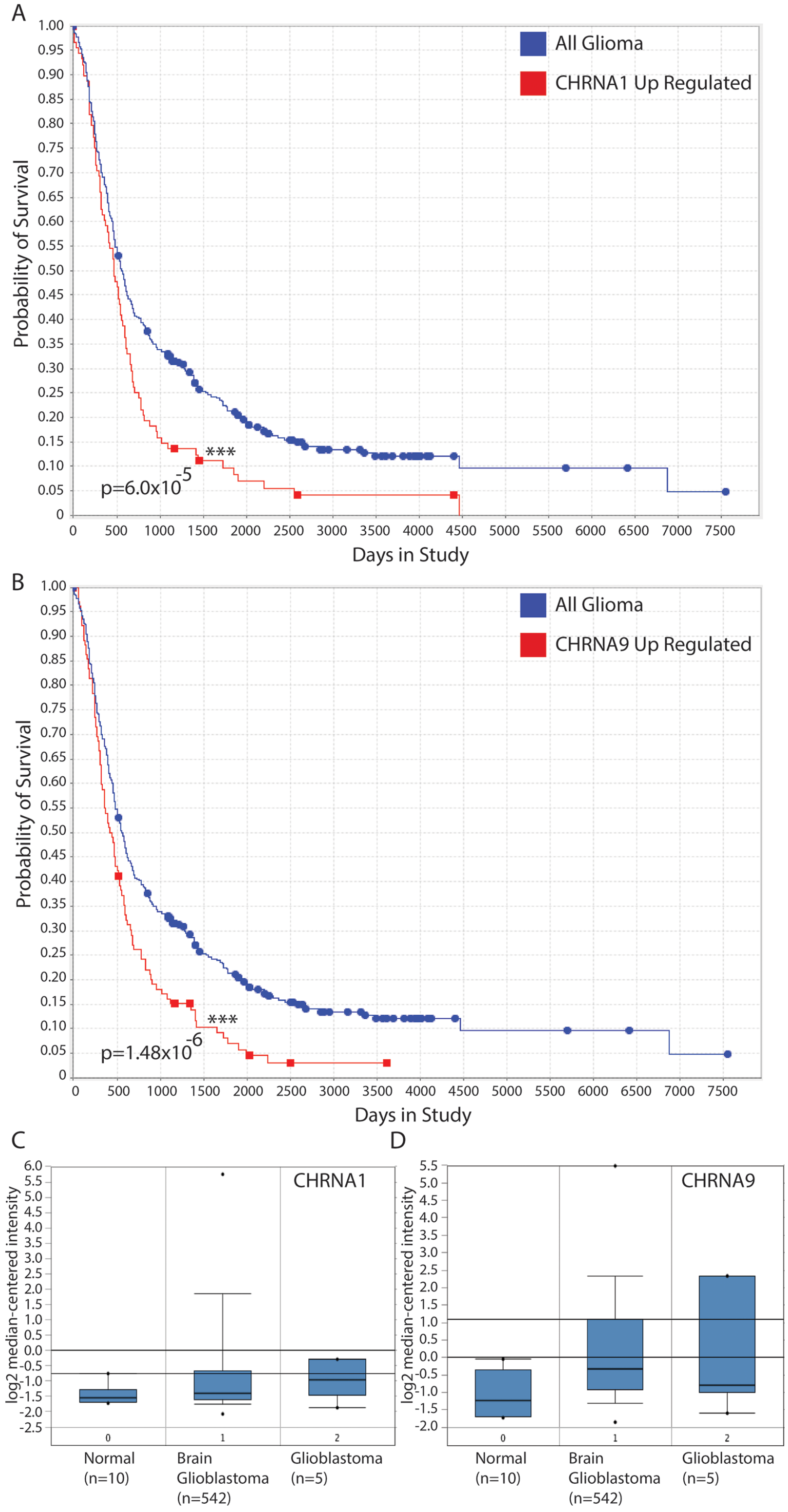

Supplementary Figure S3: Clinical relevance of AChRs expression in gliomas. Rembrandt database analysis shows that overexpression of CHRNA1 (**A**) or CHRNA9 (**B**) (encoding for AChR alpha subunits, AChRα1 and AChRα9) inversely correlates with overall survival in glioma patients $p = 6.0 \times 10^{-5}$ and $p = 1.48 \times 10^{-6}$, respectively ($n = 343$). (**C**–**D**) The Cancer Genomic Atlas (TCGA) data set on Oncomine shows overexpression of both CHRNA1 and CHRNA9 in GBM with 1.65 and 2.45 fold increases over normal controls.
